# Fear Generalization in Adolescent Anxiety Disorders: A MEG Study

**DOI:** 10.1155/da/2699511

**Published:** 2026-05-14

**Authors:** Kati Roesmann, Markus Junghöfer, Pia Kleinhölting, Thomas Straube, Ida Wessing

**Affiliations:** ^1^ Institute of Psychology, Clinical Psychology and Psychotherapy in Childhood and Adolescence, University of Osnabrück, Lise-Meitner-Str. 3, 49076, Osnabrück, Germany, uni-osnabrueck.de; ^2^ Institute for Biomagnetism and Biosignalanalysis, University Hospital Münster, Malmedyweg 15, 48149, Münster, Germany, uni-muenster.de; ^3^ Otto Creutzfeldt Center for Cognitive and Behavioral Neuroscience, University of Münster, Fliednerstr. 21, 48149, Muenster, Germany, uni-muenster.de; ^4^ Institute of Medical Psychology and Systems Neuroscience, University Hospital Münster, Von-Esmarch-Str. 52, 48149, Münster, Germany, uni-muenster.de; ^5^ Department of Child and Adolescent Psychiatry, University Hospital Münster, Schmeddingstraße 50, 48149, Münster, Germany, uni-muenster.de

**Keywords:** adolescent, anxiety disorder, EEG, fear generalization, fear conditioning, MEG

## Abstract

**Background:**

Adult patients with anxiety disorders (ADs) as well as healthy children and adolescents generalize learned fear responses to a larger degree than healthy adults. Although such overgeneralization of fear is central to theories of AD, little research has examined the respective mechanisms in pediatric populations. Therefore, we investigated behavioral and neural processes associated with fear generalization in adolescent patients with AD.

**Methods:**

Magnetoencephalography (MEG) was recorded while 30 adolescent patients with AD and matched healthy controls (HCs) performed a prevalidated fear conditioning and generalization paradigm. Differently tilted Gabor gratings served as conditioned and generalization stimuli (CS+, CS−, GS) and a screaming female face as the unconditioned stimulus (UCS). Participants rated CS and GS regarding fear and UCS expectancy. The final analyses included participants showing good data quality and contingency awareness (15 AD, 16 HC; mean age: 15.65 [±1.02], 14–17 years).

**Results:**

On the behavioral level, evidence for qualitatively different generalization patterns between groups was weak. Instead, fear and UCS‐expectancy ratings were overall higher in the AD group. On the neural level, qualitative differences emerged as a function of time. AD patients showed lower neural responses to the CS− and CS−‐like GS in frontoparietal regions (330–370 ms), while HCs showed the reverse pattern. Additionally, AD patients showed lower neural responses to the CS+ and CS+‐like GS in a sensory occipitotemporal region (>230 ms) that later showed higher responses to the CS+ (a positive gradient) in both groups (>360 ms).

**Conclusions:**

Results argue against strong qualitative differences in fear generalization in adolescent AD on a behavioral level. The observed time‐dependent qualitative differences in magnetoencephalographic responses occurred in brain regions associated with inhibitory processes (frontoparietal regions) and motivated attention (occipitotemporal regions). Further research applying temporally highly resolving electrophysiological neuroimaging appears promising to investigate the interplay of developmental and pathological generalization and its neurocognitive basis.

**Summary**

• Fear generalization is assumed to constitute a central transdiagnostic mechanism in the development of anxiety disorder (AD).
• Adults with AD show qualitatively different, for example, shallower, generalization gradients than HCs.
• Here, we found no qualitatively different generalization gradients in adolescents with AD on a behavioral level.
• On a neural level, we observed a less frontal and more posterior location of negative, presumably inhibitory gradients in all adolescents and a different timing of generalization effects in adolescents with AD.
• Further research applying temporally highly resolving electrophysiological neuroimaging seems promising to understand fear generalization processes in adolescents.

## 1. Introduction

Anxiety disorders (ADs) are among the most prevalent mental disorders [1, 2] and frequently begin during childhood and adolescence [3–5]. Influential models of AD [6, 7] indicate that the onset and maintenance of AD may occur through associative learning processes and the generalization of fear. Fear generalization, that is, the transfer of conditioned fear responses from conditioned stimuli (CS) that are associated with aversive unconditioned stimuli (UCS) to so‐called generalization stimuli (GS), can be studied in differential fear conditioning paradigms. Patterns of fear generalization along perceptual dimensions from safety‐signaling CS− via GS to threat‐signaling CS+ are characterized by generalization gradients [8, 9]. Relatively shallower, linear (vs. quadratic) gradients may indicate relatively elevated conditioned fear responses to GS, that is, overgeneralization of fear. In line with the above models, overgeneralization of fear is observed in adult patients with AD compared to healthy controls (HCs) [10, 11].

By contrast, a study investigating fear generalization in adolescents with AD (10–17 years, [12]) revealed qualitatively equivalent generalization gradients but more quantitative differences: AD patients compared to HCs rated all stimuli (CS+, CS−, and GS) as more arousing and less pleasant and indicated higher UCS expectancies. These findings question the robustness of overgeneralization as a general age‐independent marker of AD. Related to this, a recent multicenter study found no evidence for effects of self‐reported exposure to childhood adversity—that is, a risk factor for the development of ADs [13]—on generalization patterns in healthy adults [14]. Instead, and in contrast to the findings by Reinhard et al. [12], those reporting childhood adversity revealed overall blunted skin conductance responses (SCRs) and reduced CS+/CS− discrimination due to blunted SCR to the CS+, yet no differences in ratings. Taken together, these findings suggest that theories assuming overgeneralization as a pathogenic marker of ADs need refinement from a developmental perspective.

In this context, it is important that overgeneralization of fear was also linked with healthy development: Children and adolescents developed steeper gradients and thus less generalization of fear with increasing age [15–17]. Given the substantial developmental maturation processes of the neural threat‐learning circuitry [18], it seems useful to further investigate the neural mechanisms involved in both pathological and developmental fear generalization.

Research in this area has so far examined the neural basis of overgeneralization of fear in adult clinical populations (pathological overgeneralization) and healthy youths (developmental overgeneralization). Pathological overgeneralization in AD has been associated with alterations in ventromedial [10, 19] and dorsolateral [19] frontal brain regions, which may serve the inhibition of fear responses to safe stimuli. Correspondingly, higher neural responses to stimuli predicting safety (i.e., CS− and CS− like GS) compared to stimuli predicting threat (i.e., CS+) have been found in these regions, resulting in negative gradients. Moreover, fear generalization [20, 21] and pathological overgeneralization [19] have been linked to neural activity in sensory regions, which showed positive gradients emerging >300 ms after stimulus onset that likely reflect motivationally enhanced sensory processing of threat‐related stimuli. A study comparing healthy adults and adolescents replicated such negative gradients in frontoparietal and positive gradients in visual brain regions [22]. Interestingly, in the visual cluster, neural responses were overall higher in adolescents. Yet—unexpectedly, and despite qualitative differences on a behavioral level—the steepness of neural gradients in adolescents did not differ from adults. This suggests that developmental overgeneralization might not be grounded in qualitatively different neural mechanisms between healthy adolescents and adults—in contrast to pathological overgeneralization in adults.

Here, we investigated behavioral and neural correlates of fear generalization in adolescent patients with AD aged 14–18 years and matched HCs. Based on prior research [12], we predicted overall higher fear ratings and UCS expectancy ratings in adolescents with AD that might also be reflected in overall stronger neural responses to all stimuli. On both the behavioral and the neural levels, we also explored qualitative group differences, that is, we tested for differences in slopes of gradients, whereby shallower and more linear slopes would indicate pathological overgeneralization [19]. More specifically, if pathological overgeneralization in adolescents with AD depends on dynamic neural maturation processes of parts of the learning circuitry that support inhibitory functions, we would expect to see frontal brain region gradients with a shallower negative slope in AD compared to HC adolescents. Additionally, we would expect sensory brain region gradients to show shallower positive slopes in AD compared to HC adolescents.

## 2. Methods

### 2.1. Participants

Adolescent patients with AD and HC participants (14–17 years) were recruited via advertisement in schools, on social media, and in local newspapers. Patients were additionally recruited from local psychotherapists and from the Clinic for Child and Adolescent Psychiatry of the University Hospital Muenster. All participants and their parents provided written informed consent. Participants were compensated with 10 euros per hour for their participation. This study was approved by the Ethics Committee of the Medical Faculty of the University of Muenster.

Patients had to have at least one AD according to the DSM‐5. We excluded patients with comorbid disorders that we considered likely to unduly impact the conduct of the study or the study results, that is, severe depression, suicidality, bipolar disorder, psychotic disorder, obsessive–compulsive disorder, personality disorder, autism spectrum disorder, attention deficit hyperactivity disorder, posttraumatic stress disorder, anorexia and bulimia nervosa, and addictive disorder. All other comorbid disorders we included, specifically mild to moderate MDD, nightmare disorder, and binge eating disorder (see Table 1 and SM7 for details on patients’ diagnoses). HCs were excluded if they had a current or lifetime mental disorder, psychotherapy, or (psycho)pharmacological treatment. Exclusion criteria for all participants were neurological or severe somatic disease, MEG‐related exclusion criteria, pregnancy, and IQ below 80 (CFT‐20‐R, [29]). Exclusion criteria were first checked via a telephone screening with the adolescent participant and one parent. Next, the German diagnostic interview for adolescents (J‐DIPS for DSM‐IV‐TR [30, 31] and the IQ test) was conducted in person.

**Table 1:** Sample characteristics of the final sample entering behavioral and MEG analyses.

	AD	HC	Test‐statistic (df)	*p*‐Value
Gender: Female/male	14/1	15/1	*χ* ^2^(1) = 0.002	0.962
CFT^a^ (intelligence test)	99.43 (8.967)	102.19 (10.641)	*t*(28) = −0.761	0.453
Age in years	15.93 (1.163)	15.38 (0.806)	*t*(29) = 1.562	0.129
Education^a^				
School providing lower/upper secondary education	1/13	1/15	*χ* ^2^(1) = 0.010	0.922
**Psychopathology**				
Patients with one/two/three ADs	8/4/3	—	—	—
Patients with	—	—	—	—
Social anxiety disorder	12	—	—	—
Specific phobia	5	—	—	—
Agoraphobia	4	—	—	—
Panic disorder	3	—	—	—
Separation anxiety disorder	1	—	—	—
Patients with comorbid disorders	12	—	—	—
MDD (mild or moderate)	11	—	—	—
Binge eating disorder	1	—	—	—
Nightmare disorder	1	—	—	—
Pharmacological treatment				
Patients taking antidepressants	4	—	—	—
Psychometric questionnaires				
Anxiety (STAIC, DISYPS‐II)				
Trait anxiety parent‐report^b^	56.90 (10.632)	32.37 (3.774)	*t* (25.177) = 9.938	<0.001
Trait anxiety self‐report^b^	48.14 (6.937)	30.16 (5.930)	*t* (39) = 8.917	<0.001
Anxiety sympt. parent‐report^b^	36.05 (22.903)	3.00 (3.004)	*t* (20.741) = 6.552	<0.001
Anxiety sympt. self‐report^c^	39.10 (12.190)	7.22 (6.594)	*t* (37) = 9.908	<0.001
Depression (ADS‐K)^b^	23.19 (8.727)	4.85 (3.924)	*t* (28.061) = 8.747	<0.001
Intolerance of uncertainty (UI‐18)^b^				
UI‐18—total	61.1 (13.682)	34.05 (7.917)	*t* (32.329) = 7.792	<0.001
UI‐18—act	20.00 (4.990)	10.95 (3.332)	*t* (39) = 6.794	<0.001
UI‐18—burden	20.81 (5.344)	8.00 (0.725)	*t* (20.773) = 10.879	<0.001
UI‐18—vigilance	19.62 (5.408)	11.75 (3.567)	*t* (39) = 5.470	<0.001

*Note:* Gender, education, psychopathology, and pharmacological treatment are reported in absolute frequencies, and all other data are reported as mean and standard deviation (M [SD]). *T*‐tests underwent the Welch correction for unequal variances when necessary. Education is reported according to the International Standard Classification of Education (ISCED 2011). Trait anxiety was captured by the trait anxiety scale of the State–Trait Anxiety Inventory for Children STAIC, [23], German version: [24]. The presence of specific anxiety symptoms (in accordance with diagnostic criteria) was captured by the parent‐ and self‐report scales on anxiety symptoms from the German Diagnostic‐System for Mental Disorders according to ICD‐10 and DSM‐IV in Children and Adolescents (DISYPS‐II; [25]. Depression was captured by the ADS‐K [26], a German short version of the Center for Epidemiologic Depression Studies – Depression Scale, [27]. Intolerance of uncertainty was captured by the German UI‐18 (Intolerance of Uncertainty Questionnaire, [28].

^a^Data from one AD participant is missing.

^b^Data from one HC participant is missing.

^c^Data from two HC participants are missing.

We recruited 30 adolescents with AD (26 females, 4 males). Of these, six were excluded due to insufficient MEG data quality, and three qualified as outliers in the final MEG statistical analysis (see below). From a sample of healthy adolescents previously described in [22], we selected 21 HC participants that resembled the 21 AD patients regarding age, gender, IQ, and contingency awareness on a group level (for details see SM1). Finally, for our main analysis, we decided to exclude all participants without contingency awareness (see below), which resulted in a sample of 15 AD patients and 16 HC participants (Table 1).

### 2.2. Material

#### 2.2.1. Stimuli

As CS+, CS−, and GS stimuli, we used differently tilted isoluminant black‐and‐white sinusoidal gratings (Gabor gratings, see Figure 1A). The UCS was an image depicting a threatened female face presented in combination with a loud female scream. For details on stimuli and counterbalancing, see SM2.

Figure 1Experimental procedure. (A) Example stimulus set (here: set (A) with orientations between 11° and 35°; see SM2). (B) Experimental phases in chronological order. (C) Sequence of stimulus presentation during the three MEG phases. Stimuli were presented repeatedly. In the baseline phase, a warning sign predicted all UCS. In the conditioning and test phases, the CS+ predicted the UCS in 33% of the cases.

**Figure:**
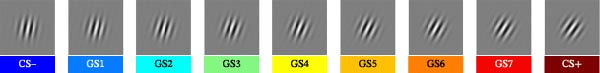
(A)

**Figure:**
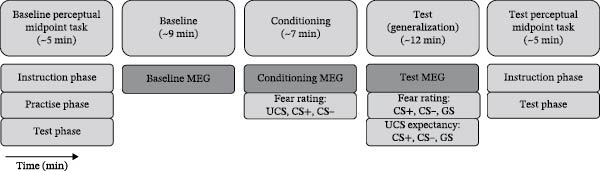
(B)

**Figure:**
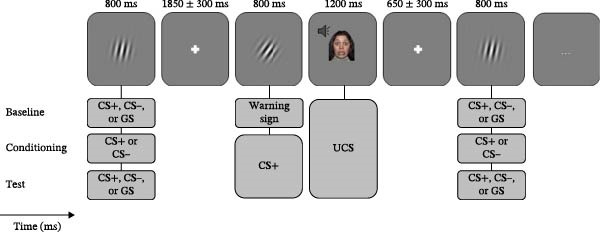
(C)

### 2.3. Experimental Procedure

#### 2.3.1. General Procedure

The experiment consisted of a baseline MEG phase, a conditioning MEG phase ending with fear ratings (CS+, CS−, and UCS), and a test MEG phase ending with fear and UCS expectancy ratings (CS+, CS−, and all GS) (Figure 1B). Before and after the three MEG phases, perceptual CS+, CS−, and GS discrimination was assessed by a perceptual midpoint (PM) task (see SM3 and Figure S1). All experimental tasks were programmed and presented using the MATLAB‐based Psychophysics Toolbox version 3 ([32]; available at www.psychtoolbox.org).

### 2.4. Generalization Paradigm

The structure of the generalization paradigm was based on an earlier fMRI study [9] and was adapted for the acquisition of event‐related fields (ERFs) [19]. The CS+, CS−, and, in the baseline and test MEG phases, the seven GS were presented separately for 800 ms in the center of the screen on a gray background (Figure 1C). During an interstimulus interval, participants were presented with either a fixation cross (1850 ± 300 ms) or the UCS (1200 ms), which was then replaced by a fixation cross (650 ± 300 ms). MEG responses to all CSs and GSs were recorded while participants focused their attention on the presented stimuli. No reactions were required.

#### 2.4.1. Baseline Phase

In the baseline phase, participants were instructed that they would see differently tilted grating stimuli and—after a “warning sign”—a screaming face (UCS). Each stimulus of one set (A–D) was presented 21 times in a pseudorandomized order. The “warning sign,” a line drawing of a triangle in black ink, was presented and replaced by the UCS seven times during the baseline phase, once in every seventh trial. As in Onat and Büchel [9], this arrangement equalized the overall arousal level in the baseline and test phases.

#### 2.4.2. Conditioning Phase

Participants were informed that they would now see two differently tilted grating stimuli, one of which would sometimes predict the screaming face. In this phase, both the CS+ and the CS− were presented 60 times each in a pseudorandomized order. In one‐third of the cases (20 times, a contingency rate of 33%), the CS+ was followed by the UCS, evenly distributed over the entire phase.

After the conditioning phase, participants rated the level of fear elicited by the CS+, the CS−, and the UCS on visual analog scales with values ranging from 1 = “no fear” to 10 = “extreme fear.”

#### 2.4.3. Test Phase

Participants were now informed that the previously learned grating stimulus would continue to predict the screaming face and that they would be presented with all the grating stimuli they knew from the baseline phase. Again, the CSs and GSs were presented pseudorandomly 21 times, while the UCS again appeared a total of seven times. However, now the CS+ (not the warning sign) predicted the UCS with a contingency rate of 33%. At the end of the test phase, the participants rated subjective fear and UCS expectancy in response to the CS+, CS−, and all GSs via button presses. The fear rating used the above visual analog scale, and the USC expectancy rating used a visual analog scale ranging from 1 = “very unlikely” to 10 = “very likely.” For both assessments, each stimulus was presented three times in a pseudorandomized order.

### 2.5. MEG Recording and Preprocessing

During the baseline, conditioning, and test phases, ERFs were acquired using a 275 MEG whole‐head sensor system (Omega 275; CTF, VSM MedTech Ltd., Coquitlam, Canada) with first‐order axial SQUID gradiometers. Continuous signals in a frequency range between 0 and 150 Hz were recorded using a sampling rate of 600 Hz. Information on the participants’ head shapes was digitized using a 3D tracking device (Polhemus, Colchester, VT, USA; www.polhemus.com). In the MEG scanner, the individual’s head position was tracked by three landmark coils placed on the two ear canals and the nasion.

The MEG data were preprocessed using the MATLAB‐based Electromagnetic Encephalography Software EMEGS (Version 3.0, [33], including offline filtering with a 48 Hz low‐pass and a 0.1 high‐pass filter and a down‐sampling to 300 Hz. Data from 200 ms before to 600 ms after stimulus onset were extracted, aligned, and baseline adjusted using a 150 ms prestimulus interval. Individual trials were edited, and artifacts were corrected using the method for statistical control of artifacts in high‐density EEG/MEG data [34]. During this process, 6 AD patients and 12 HCs were excluded from further MEG analysis because more than 30% of the trials in any run of either the baseline or the test phase did not meet our predefined data quality criteria and were, therefore, rejected (e.g., due to movement artifacts). For more details on this procedure, see Roesmann et al. [22].

The neural responses to different stimulus types were calculated separately for each participant and each phase. Each category comprised 21 trials (i.e., the number of repetitions per STIMULUS TYPE [CS+, GS1 to GS7, CS−]) in each PHASE (baseline, test). To improve the signal‐to‐noise ratio for the following estimation of underlying neural sources using the L2‐minimum‐norm estimates (L2‐MNE) method [35], ERFs of directly neighboring conditions were merged [11]. Details on these methods are outlined in SM4. To eliminate mere perceptual effects elicited by the different tilt angles of the stimuli, difference topographies (test minus baseline) of the L2‐MNE data were computed and used as the basis for statistical analyses.

### 2.6. Statistical Analyses

#### 2.6.1. Statistical Analyses of Behavioral Data

Behavioral data (ratings and PM task) were analyzed using SPSS 24 (IBM Corp., Armonk, NY). The significance level was set to *α* = 0.05 and, if necessary, *F*‐statistics were Greenhouse–Geisser corrected while *t*‐statistics were Welch corrected. To test for a potential influence of contingency awareness, all behavioral data analyses were repeated with contingency‐aware participants only. Following the definition of contingency awareness by [17], participants were considered aware of the CS–UCS relationship if UCS expectancy ratings were higher for the CS+ than the CS− and if UCS expectancy ratings for the CS− were no higher than 50%.

#### 2.6.2. Fear Ratings and UCS Expectancy Ratings

The UCS fear ratings were compared between groups (AD and HC) using an independent‐sample *t*‐test to estimate potential differences in the aversiveness of the UCS. The CS fear ratings obtained directly after the conditioning phase were analyzed by a mixed ANOVA with the within‐subject factor STIMULUS TYPE (CS+, CS−) and the between‐subject factor GROUP (AD and HC) to confirm effective fear induction in both groups. Fear ratings and UCS expectancy ratings after the test phase were analyzed by mixed ANOVAs with the within‐subject factor STIMULUS TYPE (CS+, GS1 to GS7, CS−) and the between‐subject factor GROUP (AD and HC) to determine generalization patterns to the GS stimuli. Planned polynomial contrasts tested for linear and quadratic gradients and their modulations by GROUP.

#### 2.6.3. Statistical Analyses of MEG Data

MEG data were statistically analyzed with the MATLAB‐based Electromagnetic Encephalography Software EMEGS (Version 3.0, [33]. Secondary statistical analyses used SPSS Statistics (IBM Corp., Armonk, NY). Based on evidence indicating more distinct gradients in contingency‐aware participants [17], and given that the sample characteristics did not allow us to directly test for effects of this factor, we considered contingency‐aware participants only for MEG analyses (see above). Besides this, statistical MEG data analyses paralleled the respective behavioral data analyses. To account for solely perceptual differences, all MEG analyses were based on the difference topographies (test minus baseline).

First, a linear contrast with the factor STIMULUS TYPE (CS− and GS1, GS1 and 2, GS2 and 3, GS3 and 4, GS4 and 5, GS5 and 6, GS6 and 7, GS7 and CS+) was calculated for each time point and MNE test dipole to identify spatiotemporal clusters reflecting linear generalization gradients. Second, a STIMULUS TYPE × GROUP interaction analysis with an orthogonal linear contrast for AD vs. HC was calculated for each time point and test dipole to identify clusters displaying group differences in linear gradients. This resulted in matrices of t‐values for each time point and dipole. Next, we used the nonparametric statistical cluster permutation analysis to correct for multiple comparisons [36]. This procedure determined reliably significant spatiotemporal clusters at two time intervals of interest (TOI, early: 0–300 ms, late: 300–600 ms, see Roesmann et al. [22]), with first‐ and second‐level *p* values of *p* = 0.05 (see SM5). Potential modulations of the factor GROUP were identified by secondary analyses of clusters revealing significant linear generalization gradients. For this purpose, L2‐MNE in these clusters was extracted and further analyzed by ANOVAs testing for main effects of GROUP and/or GROUP by STIMULUS TYPE interactions.

## 3. Results

Because subjective fear and UCS expectancy ratings and consequently also generalization gradients are strongly influenced by contingency awareness [17], we here report behavioral and neural effects of contingency‐aware participants (15 AD, 16 HC). Behavioral results of the complete sample (21 AD, 21 HC) are presented in the supplement (SM6).

### 3.1. Fear Ratings After Conditioning Phase

#### 3.1.1. UCS

Fear ratings in response to the UCS were marginally higher for AD patients (*M* = 5.87, SD = 2.669) than for HCs (*M* = 4.69, SD = 2.210; *t*(26.733) = 1.367, *p* = 0.128/2, one‐sided; *d* = 2.401, Figure 2A, top).

Figure 2Subjective ratings. (A) Fear rating of the UCS, CS− and CS+ at the end of the conditioning phase. (B) Fear rating of the CS−, CS+ and all GS at the end of the test phase. (C) UCS expectancy rating at the end of the test phase.

**Figure:**
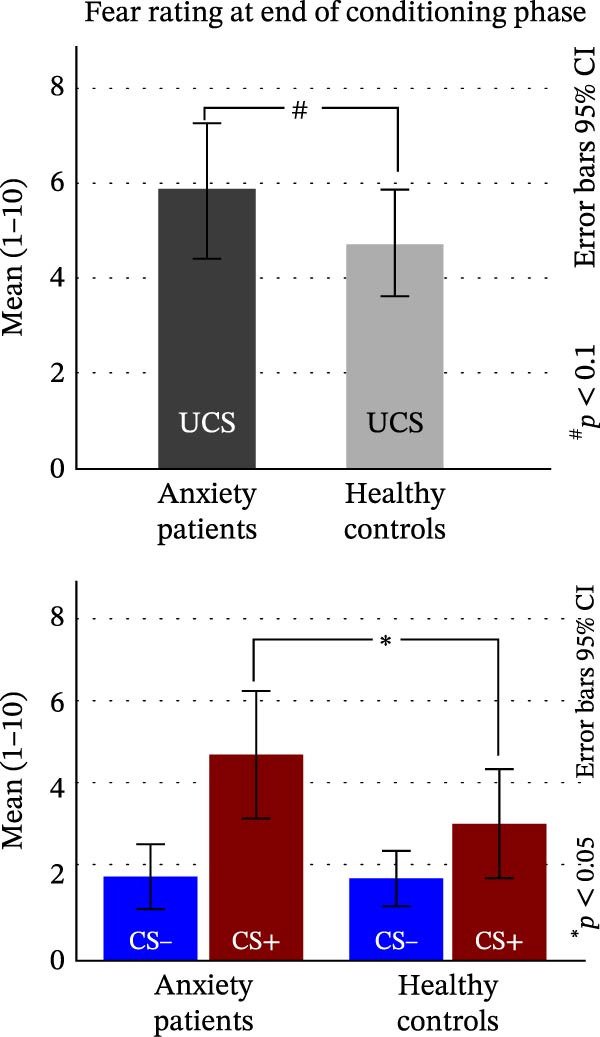
(A)

**Figure:**
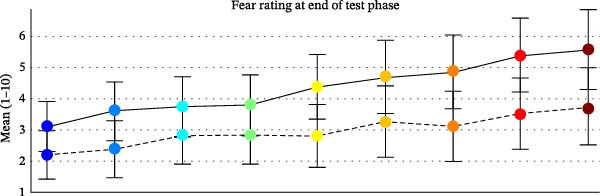
(B)

**Figure:**
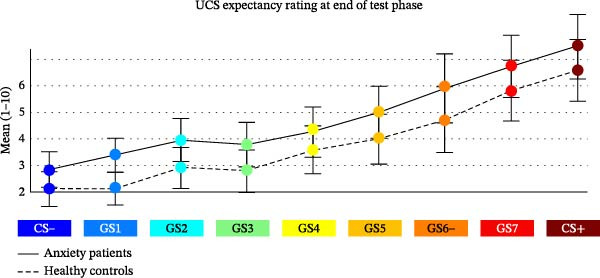
(C)

#### 3.1.2. CS

The main effect of STIMULUS TYPE (CS+, CS−) confirmed higher fear ratings in response to the CS+ vs. the CS− (*F*(1, 29) = 38.154, *p* < 0.001, *η* ^2^ = 0.568; Figure 2A, bottom). A main effect of GROUP indicated marginally higher fear ratings in AD vs. HC (*F*[1, 29] = 1.820, *p* = 0.188/2, one sided, η^2^ = 0.059). A significant STIMULUS TYPE × GROUP interaction (*F*[1, 29] = 5.842, *p* = 0.022, *η* ^2^ = 0.168) indicated marginally higher fear ratings of the CS+ in the AD vs. HC group (*t*(28.296) = 1.842, *p* = 0.076, *d* = 2.623), while fear ratings of the CS− did not differ between groups (*t*(27.906) = 0.110, *p* = 0.457, *d* = 1.367).

### 3.2. Fear Ratings After Test Phase

#### 3.2.1. CS and GS

For fear ratings (Figure 2B), the ANOVA with the factors STIMULUS TYPE and GROUP revealed a significant main effect of STIMULUS TYPE (*F*[3.266, 94.711] = 26.487, *p* < 0.001, *η* ^2^ = 0.477), which was characterized by a linear positive gradient (*F*[1, 29] = 57.090, *p* < 0.001, *η* ^2^ = 0.663). A main effect of GROUP revealed overall higher fear ratings for the AD vs. HC group (*F*[1, 29] = 4.083, *p* = 0.053/2, one‐sided, *η* ^2^ = 0.123). The GROUP × STIMULUS TYPE interaction was marginally significant (*F*[3.266, 94.711] = 2.340, *p* < 0.073, *η* ^2^ = 0.075). The linear increase of fear ratings from the CS− to the CS+ was (marginally) steeper in the AD compared to the HC group (*F*[1, 29] = 4.031, *p* = 0.054; *η* ^2^ = 0.122).

#### 3.2.2. UCS Expectancy Ratings After Test Phase

For UCS expectancy ratings (Figure 2C), we found a significant main effect of STIMULUS TYPE (*F*[1.955, 78.186] = 30.640, *p* < 0.001, *η* ^2^ = 0.434), which was characterized by a linear (*F*[1, 29] = 107.996, *p* < 0.001, *η* ^2^ = 0.788) and a quadratic gradient *F* (1, 29) = 27.049, *p* < 0.001, *η* ^2^ = 0.483).

The main effect of GROUP revealed overall higher UCS expectancy ratings in the AD vs. HC (*F*[1, 29] = 3.136, *p* = 0.087/2, one‐sided, *η* ^2^ = 0.098). The STIMULUS × GROUP interaction was not significant (*F*[2.404, 69.730] = 0.228, *p* = 0.987, *η* ^2^ = 0.008).

### 3.3. Changes of MEG‐Based Estimated Neural Activity From Baseline to Test Phase

#### 3.3.1. Group‐Independent Linear Contrasts

##### 3.3.1.1. Early Time Interval (0–300 ms)

**Figure 3:**
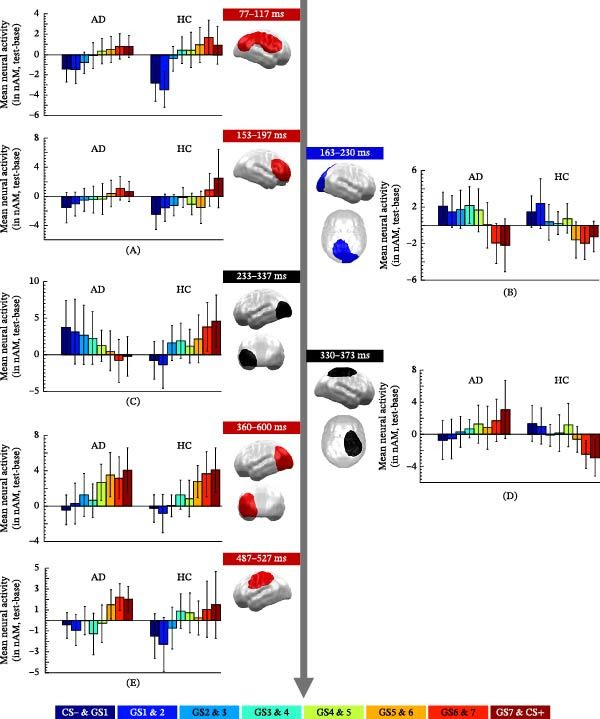
MEG results. (A) Positive (red) linear gradients in the early TOI. (B) Negative (blue) linear gradients in the early TOI. (C) Orthogonal (black) linear contrasts with positive gradients in HCs in the early TOI, extending to the late TOI. (D) Orthogonal (black) linear contrasts with negative gradients in HCs in the late TOI. (E) Positive (red) linear gradients in the late TOI.

Within the early TOI (Figure 3A), we found three spatiotemporal clusters showing positive linear gradients. Two clusters were merged, as they appeared in largely overlapping time intervals (77–117 ms, *p*‐cluster = 0.003; 77–110 ms, *p*‐cluster = 0.018) and adjacent right ventral occipitoparietal areas that extended to frontal sites. In addition to the linear gradient (*F*[1, 29] = 18.807, *p* < 0.001, *η* ^2^ = 0.393), the merged cluster (77–117 ms) also revealed a quadratic gradient (*F*[1, 29] = 4.661, *p* < 0.024, *η* ^2^ = 0.163). Neither linear nor quadratic effects were modulated by the factor GROUP (*p*’s > 0.173). The third cluster emerged slightly later in right ventrolateral prefrontal regions (153–197 ms, *p*‐cluster: 0.029 (linear gradient: *F*[1, 29] = 7.883, *p* = 0.009, *η* ^2^ = 0.214; quadratic gradient: n.s, *p* = 0.492). Again, both gradients were not modulated by the factor GROUP (*p*’s > 0.373).

In a temporally overlapping time interval, we observed two clusters with negative linear gradients in adjacent occipital and parietal brain regions (163–230 ms, *p*‐cluster = 0.002; 180–220 ms, *p*‐cluster = 0.014) that were merged into one cluster (Figure 3B). This cluster showed a negative linear gradient (*F*[1, 29] = 18.067, *p* < 0.001, *η* ^2^ = 0.384) but no quadratic gradient (*F*[1, 29] = 2.193, *p* = 0.149, *η* ^2^ = 0.070), and both gradients were again not modulated by the factor GROUP (*p*’s > 0.122).

##### 3.3.1.2. Late Time Interval (300–600 ms)

In the late TOI (Figure 3E), we observed four temporally and spatially largely overlapping clusters, which showed the hypothesized positive gradients in left ventral occipital brain regions and were thus merged (360–600 ms) for visualization purposes and for further analyses (linear gradient: *F* [1, 29] = 18.060, *p*  < 0.001, *η* ^2^ = 0.384; quadratic gradient: n.s, *p* = 0.647). Gradients were again not modulated by the factor GROUP (*p*’s > 0.488). Qualitatively similar effects were revealed in left parietal regions (487–527 ms, *p*‐cluster = 0.019, linear gradient: *F* [1, 29] = 8.872, *p* = 0.006, *η* ^2^ = 0.234, quadratic gradient: *F* [1, 29] = 0.398, *p* = 0.533, *η* ^2^ = 0.234). While the linear gradient was again not modulated by GROUP (F[1, 29] = 0.005, *p* = 0.946, *η* ^2^ < 0.001), the quadratic gradient showed a marginal interaction with GROUP (*F*[1, 29] = 3.450, *p* = 0.073, *η* ^2^ = 0.106). However, post hoc analyses of quadratic gradients in the AD and HC groups separately revealed no significant results (*p*’s > 0.146).

No (group‐independent) negative linear gradients were observed in the late TOI.

#### 3.3.2. Orthogonal Linear Contrasts Indicating Qualitative Group Differences

##### 3.3.2.1. Early Time Interval (0–300 ms)

Analyses testing for orthogonal linear contrasts (i.e., qualitative group differences in linear gradients) in the early TOI (Figure 3C) revealed one significant cluster (233–337 ms, *p*‐cluster = 0.045). It was located in the left ventro‐occipital brain regions, that is, in brain regions that revealed group‐independent positive gradients at later time intervals (Figure 3E). Group differences between linear gradients (*F*[1, 29] = 16.917, *p* < 0.001, *η* ^2^ = 0.368) were characterized by positive gradients for HCs (*F*[1, 15] = 8.019, *p* = 0.013, *η* ^2^ = 0.348), while patients with AD revealed negative gradients (*F*[1, 14] = 9.752, *p* = 0.007, *η* ^2^ = 0.411) in this cluster.

##### 3.3.2.2. Late Time Interval (300–600 ms)

Additionally, analyses for orthogonal linear contrasts in the late TOI (Figure 3D), yielded one significant cluster in right parietal regions that extended to dorsal frontal sites (330–373 ms, *p*‐cluster = 0.017). The orthogonal contrast (*F*[1, 29] = 11.192, *p* = 0.002, *η* ^2^ = 0.278) was characterized by negative linear gradients in HCs (*F*[1, 15] = 2.855, *p* = 0.009, *η* ^2^ = 0.160), but positive linear gradients in AD (*F*[1, 14] = 2.220, *p* = 0.039, *η* ^2^ = 0.137). Thus, significant orthogonal contrasts in left sensory (Figure 3C) and right parietal (Figure 3D) clusters revealed gradients in opposite directions.

## 4. Discussion

In this study, we investigated behavioral and neural correlates of fear generalization in adolescent patients with AD. Supporting evidence by Reinhard et al. [12], we found overall higher fear ratings and UCS expectancy ratings of CS and GS as well as marginally higher fear ratings of the UCS in patients with AD. While group effects were only marginally significant after the conditioning phase, they reached significance after the test phase in both fear and UCS expectancy ratings. Yet, despite stable generalization effects supporting the validity of the paradigm, evidence for qualitatively different generalization gradients between groups (i.e., shallower or more linear slopes indicating overgeneralization indexed by interactions of the factors group and stimulus) was weak on the behavioral level. The results clearly speak against a reduced CS+/CS− discrimination in adolescent patients with AD, in contrast to findings in healthy adult individuals with a history of adverse childhood experiences [14]. This observed discrepancy may indicate that certain patterns of associative learning that are related to childhood experiences might even constitute a protective factor preventing the development of psychopathology.

On a neural level, we found more negative gradients in HC than in AD participants in frontoparietal networks (330–373 ms, Figure 3D). The timing of this effect and, partly, its spatial characteristics reflect our previous findings in both healthy adults and healthy adolescents (partly also reported here) [22] revealing negative gradients in dorsal frontal networks. We previously suggested that these negative gradients reflect (adaptive) inhibition of CS− and CS− like GS, as the procedure of differential fear conditioning might add an inhibitory component to the CS− [37]. In line with the assumption that impaired inhibitory responses to safe stimuli are a pathogenic characteristic of AD, adolescent AD patients did not show a negative gradient but, in fact, even showed a positive gradient in the identified frontoparietal cluster. Interestingly, generalization effects in similar (yet left‐hemispheric) frontoparietal networks and with comparable timing have been shown to be affected by excitatory vs. inhibitory noninvasive transcranial stimulation of the vmPFC [38]. Specifically, healthy adults revealed negative gradients (like healthy adolescents here) after sham and excitatory stimulation of the vmPFC, while they showed positive gradients (like adolescent AD patients here) after inhibitory vmPFC stimulation, suggesting that experimental inhibition of the vmPFC might serve as a model for pathological overgeneralization [38]. If the functionality of prefrontal structures is reduced (e.g., by inhibitory brain stimulation or due to neural immaturity), inhibitory prefrontal functions may be adopted by parietal brain structures. Indeed, in contrast to our previous studies in adults [19, 38, 39], adolescents generally showed negative gradients at more posterior but not frontal sites (Figure 3B), which aligns with a developmental shift of cognitive task‐related neural activation patterns from posterior to frontal regions [40].

In addition, we found a reverse pattern of interaction effects in sensory occipitotemporal brain regions (233–337 ms, Figure 3C). Like spider‐phobic adults who responded to later exposure therapy [19], healthy adolescents showed a relatively early positive gradient in visual regions. By contrast, like non‐responders to exposure therapy, adolescent AD patients showed a distinct negative gradient in this cluster. Interestingly, this effect was followed by the predicted positive generalization gradient—now in both groups—in the same region (>360 ms. Figure 3E). Convergent findings of positive gradients in widespread brain networks including occipitotemporal brain regions nicely align with previous research in adults [19–21, 38, 41]. Several ERP/ERF studies revealed generalization effects in the time window of the late positive potential (LPP), starting around 300 ms after stimulus onset. This component has been functionally linked to prioritizing the perceptual processing of emotionally salient stimuli [42]. Consistent results were also observed in adolescents, who showed enhanced LPP amplitudes in response to threat‐signaling CS+ compared to CS− during both fear acquisition [43] and extinction recall [44]. In the present study, the occurrence of positive gradients in sensory regions, which arguably reflect a prioritized processing of motivationally relevant threat stimuli, was apparently delayed in adolescent AD patients compared to HCs.

Our study suggests that the spatiotemporal sequence of generalization gradients may be crucial for identifying neural generalization patterns that characterize AD in adolescents. How fast certain brain regions are activated may be more relevant than whether they are activated (see also [45]).

What are the clinical implications of these findings? In light of rather weak evidence for behavioral group differences in generalization patterns, functional interpretations or clinical implications of differences in neural generalization patterns remain highly speculative: following our line of argument that the reported early spatiotemporal alterations in generalization patterns of patients with AD may reflect early signatures of emotional attention, our findings point to the potential of bottom‐up interventions designed to alter perceptual and attentional stages of affective processing, for example, perceptual discrimination trainings (PDC) or attention bias modification (ABM). PDC was shown to reduce overgeneralization in ratings of healthy adults [46] and in physiological measures – but not in ratings – in children [47]. Yet, putative effects on clinical symptoms were not addressed in these studies. Likewise, a systematic review and meta‐analysis on ABM in youth [48] suggests that this approach may reliably alter attention bias metrics. However, mixed evidence for clinically relevant effects and sometimes low quality of the primary studies included in this meta‐analysis [48] demand caution regarding the clinical effectiveness of behavioral bottom‐up approaches to treat anxiety in youth. Future experimental research should clarify to what degree bottom‐up behavioral approaches, like PDC or ABM, or biological approaches like targeted non‐invasive brain stimulation (e.g., [38]) may change early spatiotemporal alterations in neural generalization patterns of patients with AD and whether such approaches may yield beneficial effects on clinical symptoms.

### 4.1. Limitations

Findings should be interpreted in the light of several limitations. First, a rather small sample entered our main analyses. Our pre‐validated generalization paradigm was challenging for adolescents, particularly for those with AD. This led to exclusions due to insufficient MEG data quality. Moreover, although the applied contingency rate of 33% is recommended to detect qualitative differences between healthy and AD participants [49], it came at the cost of a rather high ratio of contingency‐unaware participants (see Figure S1). Future studies should directly investigate the influence of contingency awareness on generalization in AD.

Second, despite a successful matching of age, gender, IQ, and contingency awareness, our groups differed not only in their level of anxiety but also in their level of depression and intolerance of uncertainty. Additionally, the AD group included different types of AD and comorbid disorders. Though such heterogeneity reflects the norm and allows for generalizability, in this small sample, it may be the source of confounds. While models of AD assume fear generalization to be a transdiagnostic marker, fine‐grained age‐ and disorder‐specific aspects [50] as well as the consideration of relevant life events [14, 51] seem relevant to understanding the interplay of developmental and pathological overgeneralization as well as the development of anxious psychopathology more broadly. Additionally, as discussed above, further research is needed to link specific neural mechanisms with clinical markers of anxiety. Effects of mechanism‐informed interventions on basic processes as well as on clinical outcomes may be informative in the ultimate goal to bridge the existing translational gaps.

## 5. Conclusion

In the light of these limitations, our findings should be considered a first step to understand the neurocognitive basis of generalization processes in adolescents with AD. They do not argue for strong qualitative differences in fear generalization, at least on a behavioral level. The observed qualitative differences in magnetoencephalographic responses across time and their spatial characteristics suggest that inhibitory processes, particularly in frontoparietal networks, and emotional attention, particularly in sensory networks, might differ between groups. To understand the interplay of developmental and pathological generalization processes in adolescents, further research applying temporally highly resolving electrophysiological neuroimaging seems promising.

## Funding

This work was funded by the Deutsche Forschungsgemeinschaft (Grants SFB‐TRR58‐C07 JU‐445/9‐1, SFB‐TRR393‐A04 and SFB‐TRR393‐B07) and the Innovative Medizinische Forschung (Grant RO211907). Open Access funding enabled and organized by Projekt DEAL.

## Conflicts of Interest

The authors declare no conflicts of interest.

## Supporting Information

Additional supporting information can be found online in the Supporting Information section.

## Supporting information


**Supporting Information** Figure S1: Perceptual midpoint (PM) task. SM1: Table showing demographic and learning‐related variables in the AD and HC groups and description of the matching process. SM2: Additional details of the experimental design. SM3: Perceptual midpoint task. SM4: Additional details of the MEG analyses. SM5: Cluster permutation analysis. SM6: Results of the full sample, including contingency‐unaware adolescents. SM7: Diagnoses of individual AD patients.

## Data Availability

The data that support the findings of this study are openly available in G‐Node at https://doi.gin.g-node.org, reference number https://doi.org/10.12751/g-node.7155v7.
