# Supplementary material for: Fear Generalization in Adolescent Anxiety Disorders: A MEG Study
Source: Depress Anxiety. 2026 May 14;2026:2699511. doi: 10.1155/da/2699511 (PMC13176784; doi:10.1155/da/2699511)
Supplement: Supplementary file 1 — Supporting Information Figure S1: Perceptual midpoint (PM) task. SM1: Table showing demographic and learning‐related variables in the AD and HC groups and description of the matching process. SM2: Additional details of the experimental design. SM3: Perceptual midpoint task. SM4: Additional details of the MEG analyses. SM5: Cluster permutation analysis. SM6: Results of the full sample, including contingency‐unaware adolescents. SM7: Diagnoses of individual AD patients. [file DA-2026-2699511-s001.docx]

**Supplementary Materials**

## Fear Generalization in Adolescent Anxiety Disorders: A MEG Study

**Authors:**

Kati Roesmann, PhD*^1,2,3^, Ida Wessing, PhD* ^2,3,4^, Pia Kleinhölting^4^, Thomas Straube^5^, PhD & Markus Junghöfer, PhD^2,3^

^1^ Institute of Psychology, Clinical Psychology and Psychotherapy in Childhood and Adolescence, University of Osnabrück, Lise-Meitner-Str. 3, 49076 Osnabrück, Germany

^2^ Institute for Biomagnetism and Biosignalanalysis, University Hospital Münster, Malmedyweg 15, 48149 Münster, Germany

^3^ Otto Creutzfeldt Center for Cognitive and Behavioral Neuroscience, University of Münster, Fliednerstr. 21, 48149 Muenster, Germany

^4^ Department of Child and Adolescent Psychiatry, University Hospital Münster, Schmeddingstraße 50, 48149 Münster, Germany

^5^ Institute of Medical Psychology and Systems Neuroscience, University Hospital Münster, Von-Esmarch-Str. 52, 48149 Münster, Germany

*equal contribution

Corresponding Author:

Ida Wessing

Department of Child and Adolescent Psychiatry

University Hospital Münster

Schmeddingstraße 50

48149 Münster, Germany

Phone: +49 176 31258119

Email: Ida.Wessing@ukmuenster.de

## SM1

**Table S1:** Comparison of key demographic (age, gender) and learning-related (IQ, contingency awareness) variables observed in the AD patients (N = 30) and the total available HC group (N = 61, as reported in (Roesmann, Wessing, et al., 2022)).

|  | AD | HC | Test-statistic (df) | p-value |  |
| --- | --- | --- | --- | --- | --- |
| Gender: Female / Male (N) | 26/4 | 32/28 | χ *^2^*(1) = 9.698 | .002 |  |
| Contingency Awareness: aware / non aware (N) | 22/8 | 54/6 | χ*^2^*(1) = 4.229 | .040 |  |
| CFT (M(SD)) | 100.21 (10.884) | 110.02  (13.099) | t(84) = -3.467 | < .001) |  |
| Age in years (M(SD)) | 15.73 (1,143) | 15.63  (1,073) | t(88)= 0.408 | .684 |  |

**Matching Process:**

As can be seen in Table S1, the distribution of gender, contingency awareness and IQ systematically differed between the AD group and the sample of HCs reported in (Roesmann, Wessing, et al., 2022). As all of these variables have previously been linked with associative learning and/or fear generalization (age & contingency awareness: (Schiele et al., 2016), gender: (Roesmann, Leehr, et al., 2022)), they were regarded potential confounds in the attempt to investigate the effect of pathological anxiety on fear generalization. Therefore, for the study at hand, we selected a subsample of HC participants that matched the AD group regarding age and gender as well as IQ and contingency awareness. In this process, participants with insufficient MEG data quality (see SM4) were not considered.

## SM2 Experimental Design

All participants received written and verbal information on the generalization paradigm. Next, participants entered the soundproof and magnetically shielded MEG chamber and were placed in the MEG scanner at a distance of 90 cm from the monitor.

In total, the study took about two hours.

### Conditioned and Generalization Stimuli (CS and GS)

As conditioned (CS) and generalization (GS) stimuli, we used isoluminant black-and-white sinusoidal grating stimuli of different tilt angles that were filtered with a Gaussian envelope and had a maximum Michelson contrast of 95% (see Figure 1A main text). We used four sets of nine stimuli each, whereby the orientation of neighboring stimuli differed by 3°. Prominent orientations (0°, 45°, 90°, 135°) were excluded. The final sets included stimuli with orientations between 11° and 35° (set A), 101° and 125° (set B), 56° and 80° (set C) and 146° and 170° (Set D). The two most divergent stimuli in each set (differing by 24°) were used as CS+ and CS- stimuli, and the seven stimuli in between were the generalization stimuli (GS). The assignment of orientations to CS+ and CS- was balanced across participants.

### Unconditioned Stimuli

The unconditioned stimulus was a picture depicting a threatened female face (NimStim Face Stimulus Set; Tottenham et al., 2009) in combination with a female scream from the International Affective Digitized Sounds System (Bradley & Lang, 1999). The scream was presented at a sound pressure level of 60 dB above the participants' individual hearing threshold, which corresponds to approximately 95 dB (range 85 to 105 dB); t-tests comparing the sound pressure levels of AD vs. HC participants were not significant for the right and left and the mean of both ears all t < 1.

### SM3: Perceptual Midpoint Task

#### Design

The PM task was adapted from research on mechanisms of perceptual learning (McMahon & Leopold, 2012) and was used previously (Roesmann, Kroker, et al., 2022; Roesmann, Leehr, et al., 2022; Roesmann, Wessing, et al., 2022). Performance in the PM task was measured before (baseline PM task) and after (test PM task) the conditioning and generalization phase (see Figure 1A) and served as an index for different aspects of perceptual discrimination. In the task, the CS+ and CS- stimuli were positioned on the left and right side of a screen (Right-CS+/Left-CS- or Left-CS+/Right-CS- assignment alternated between participants), and participants had to indicate via a forced-choice button press whether a centrally presented GS would be more similar to the CS+ or the CS-. For more details, see (Roesmann, Kroker, et al., 2022).

#### Statistical analyses

The relative frequency of classifications as CS+ was determined separately for each PHASE (baseline PM task, test PM task) and for each GS. A mixed ANOVA with the within-subject factors STIMULUS TYPE (GS1 to GS7) and PHASE (Baseline, Test) and the between-subject factor GROUP (AD, HC) was computed. Planned polynomial contrasts tested for linear and quadratic and cubic trends and their modulations by PHASE and GROUP. Based on the conception of the PM task, shifts of the perceptual midpoint should be reflected as changes of cubic trends (Roesmann, Kroker, et al., 2022). Group-specific patterns were further investigated using repeated-measures ANOVAs with the within-subject factors STIMULUS TYPE (GS1 to GS7) and PHASE (Baseline, Test) for AD patients and HCs separately. Planned polynomial contrasts tested for linear and quadratic trends and their modulations by PHASE.

#### Results: Changes in the perceptual midpoint (PM) task from baseline to test phase

Statistical analyses of the PM task revealed a significant main effect of STIMULUS TYPE (F(2.726, 106.304) = 258.606, p < .001, η2 = 0.869; linear: F(1, 39) = 1010.741, p < .001, η2 = 0.963; cubic: F(1, 39) = 29.751, p < .001, η2 = 0.433) and a significant interaction of STIMULUS TYPE x PHASE (F(4.913, 101.597) = 3.312, p = .007, η2 = 0.078; linear: F(1, 39) = 4.323, p = .044, η2 = 0.100; quadratic: F(1, 39) = 6.438, p = .015, η2 = 0.142. Conceptually, this interaction was characterized by steeper gradients in the test vs. the baseline PM task, which indicates a better discrimination in the test phase. Additionally, we observed a main effect of PHASE (F(1, 39) = 4.510, p = .040, η2 = 0.104), which indicated an overall higher perceived similarity with the CS+ in the test PM task than in the baseline PM task.

Importantly, these effects were not modulated by the factor GROUP (STIMULUS TYPE x GROUP: F(2.726, 106.304) = 0.500; p = .665; η2 = 0.013; STIMULUS TYPE x PHASE x GROUP: F(4.913, 191.597) = -1.085; p = .370; η2 = 0.027; PHASE x GROUP: F(1, 39) = .011, p = .917, η2 = 0.000). Thus, AD and HC showed equivalent overall and learning-related discrimination performance. Note that data from one contingency-aware AD participant was missing.

Results were largely replicated when considering the contingency-aware group only (Figure S1). Again, we observed a significant main effect of STIMLUS TYPE (F(2.542, 71.206) = 188.164, p < .001, η2 = 0.870; linear: F(1, 28) = 747.394, p < .001, η2 = 0.964; cubic: F(1, 28) = 23.081, p < .001, η2 = 0.452) and a significant interaction of STIMULUS TYPE x PHASE (F(4.448, 124.540) = 2.557, p = .036, η2 = 0.084; quadratic: F(1, 28) = 8.476, p = .007, η2 = 0.234; Note, now linear effects were only marginally significant: F(1, 28) = 2.898, p = .100, η2 = 0.094, while cubic trends were non-significant: F(1, 28) = 1.409, p = .245, η2 = 0.048). There was no main effect of PHASE (F(1, 28) = 2.085, p = .165, η2 = 0.068).

Again, these effects did not differ between AD patients and HCs (STIMULUS TYPE x GROUP: F(2.543, 71.206) = 0.893, p = .435; η2 = 0.031, STIMULUS TYPE x PHASE x GROUP: F(4.448, 124.540) = 1.105, p = .360, η2 = 0.038, PHASE x GROUP: F(1, 28) = 0.285, p = .597, η2 = 0.010).

**Figure S1: Perceptual Midpoint Task**

**
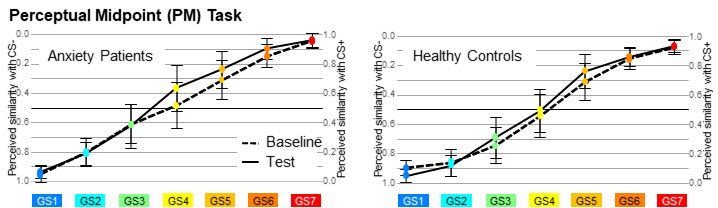
**

Figure Legend

## SM4: Signal Averaging, L2-Minimum Norm Estimation and Artifact Rejection

Each category comprised 21 trials (i.e., the number of repetitions per STIMULUS TYPE (CS+, GS1 to GS7, CS-)) in each PHASE (baseline, test). This rather low number of trials per condition resulted from the need to keep the runs as short as possible to avoid movements, reduced vigilance and poor attention performance. To increase the number of trials per category, ERFs in response to CS+ were merged with those to GS1 (CS+/GS1), those to GS1 with those to GS2 (GS1/GS2), and so on (Lissek et al., 2010). This moving average doubled the number of trials per step and, thus, improved the signal-to-noise ratio by about 40% (√2×100%). This was particularly important to get a reasonable estimate of the underlying neural sources. In addition, the high-frequency noise along the vector of the STIMULUS TYPE was reduced, while the resolution of the STIMULUS TYPE gradient function was reduced from nine to only eight steps.

The L2-MNE is an inverse modeling technique that can be used to estimate the activity of distributed neural networks. It does not require a priori information on the location and/or number of active current dipoles (Hauk, 2004). The source model used was a spherical shell with 350 evenly spaced dipole pairs (azimuthal and polar directions) with a source shell radius approximately equal to the gray matter depth (i.e., 87% of the individualized head). The Tikhonov regularization parameter Lambda was set to 0.1. The topographies of source direction-independent neuronal activity – the vector length of estimated source activity at each position – were calculated for each individual participant, condition and time point. To avoid statistical artifacts due to potential outliers in any phase or condition, participants were excluded if the mean of the standard deviation between experimental conditions across time (2 AD, 2 HC) or the mean number of trials across experimental conditions differed from the median of the sample by more than four standard deviations (1 AD; 1 HC was also excluded due to the other outlier criterion). The number of remaining trials was evenly distributed across the experimental conditions, as confirmed by an 8 x 2 x 2 ANOVA with the factors STIMULUSTYP (CS-&GS1, GS1&2, GS2&3, GS3&4, GS4&5, GS5&6, GS6&7, GS7&CS+), PHASE (Baseline, Test) and GROUP (AD, HC) with all p > .113.

## SM 5: Cluster Permutation Analysis

In each of the two TOIs (0-300 ms and 300-600 ms), t-values exceeding the critical alpha level of p =.05 (first or sensor level criterion) entered so-called spatiotemporal cluster masses (spatio-temporal integral of t-values within each cluster). Cluster masses were then compared against identical analyses based on 1,000 permuted drawings of data from the experimental conditions. When the cluster mass of the original analysis (using the correct assignment of experimental conditions) was higher than the critical cluster mass of this permutation distribution corresponding to a p-value = .05 (i.e., higher than the 950th biggest cluster masses found with the largest cluster of each draw of the random distribution; second or cluster level criterion), the cluster was considered significant. If any effect overlapped the interval limit of 300 ms, the interval was extended stepwise by 50 ms (i.e. 0-350 ms or 250-600 ms etc.).

## SM6: Results of the full sample including contingency unaware adolescents (21 AD, 21 HC)

## Fear Ratings after Conditioning Phase

## UCS

Fear ratings in response to the UCS were higher for AD patients (M = 6.24, SD = 2.528) than for HCs (M = 4.29, SD = 2.148; t(38.984) = 2.697, p *=*.01/2, one-sided; d = 2.346).

## CS

A main effect of STIMULUS TYPE (CS+, CS-) revealed higher fear ratings in response to the CS+ vs. the CS- (F(1, 40) = 24.475, p < .001, η^2^ = 0.380). A main effect of GROUP indicated higher fear ratings in AD vs. HC (F(1, 40) = 5.342, p = .026/2, one-sided, η^2^ = 0.118). The interaction STIMULUS TYPE x GROUP was non-significant (F(1, 40) = 2.411, p *=*.126, η^2^ < 0.058).

## Fear Ratings after Test Phase

## CS and GS

For fear ratings, the ANOVA with the factors STIMULUS TYPE and GROUP revealed a significant main effect of STIMULUS TYPE (F(2.98, 119.074) = 21.762, p <.001, η^2^ = 0.352), which was characterized by a linear positive trend (F(1, 40) = 41.216, p < .001, η^2^ = 0.507). A main effect of GROUP revealed overall higher fear ratings for the AD vs. HC group (F(1, 40) = 3.601, p = .065/2, one-sided, η^2^ = 0.083). The interaction of STIMULUS TYPE x GROUP was not significant (F(2.98, 119.074) = 0.774, η^2^ = 0.019).

## UCS Expectancy Ratings after Test Phase

For UCS expectancy ratings, we found a significant main effect of STIMULUS TYPE (F(1.955, 78.186) = 30.640, p <.001, η^2^ = 0.434), which was characterized by a linear (F(1, 40) = 42.925, p < .001, η^2^ = 0.518) and a quadratic trend (F(1, 40) = 17.248, p < .001, η^2^ = 0.301). The main effect of GROUP when testing for overall higher UCS expectancy ratings in the AD vs. HC group was only marginally significant (F(1, 40) = 2.232, p = .143/2, one-sided, η^2^ = 0.053). The interaction of STIMULUS TYPE x GROUP was not significant (F 1.955, 78.186) = 0.323, η^2^ = 0.008).

**SM7: Diagnoses of individual AD patients**

| Patient | CA | Diagnosis 1 | Diagnosis 2 | Diagnosis 3 | Diagnosis 4 |
| --- | --- | --- | --- | --- | --- |
| 1 | yes | Specific Phobia |  |  |  |
| 2 | yes | Social AD |  |  |  |
| 3 | yes | Panic Disorder | Separation AD | Specific Phobia | Nightmare Disorder |
| 4 | yes | Social AD | Specific Phobia | MDD |  |
| 5 | yes | Agoraphobia | Panic Disorder | Social AD | MDD |
| 6 | yes | Social AD | MDD |  |  |
| 7 | yes | Social AD | MDD |  |  |
| 8 | yes | Agoraphobia | Social AD | MDD |  |
| 9 | yes | Agoraphobia | Social AD | Specific Phobia | MDD |
| 10 | yes | Social AD |  |  |  |
| 11 | yes | Social AD | MDD |  |  |
| 12 | yes | Social AD | MDD |  |  |
| 13 | yes | Social AD | Specific Phobia | MDD |  |
| 14 | yes | Agoraphobia | Panic Disorder | MDD |  |
| 15 | yes | Social AD | MDD | Binge Eating Disorder |  |
| 16 | no | Agoraphobia | Social AD | MDD |  |
| 17 | no | Social AD | Specific Phobia |  |  |
| 18 | no | Social AD | MDD |  |  |
| 19 | no | Specific Phobia |  |  |  |
| 20 | no | Agoraphobia | Social AD | MDD |  |
| 21 | no | Social AD | MDD |  |  |

## References

Bradley, M.M., Lang, P.J., 1999. International affective digitized sounds (IADS): Stimuli,

instruction manual and affective ratings. Technical Report B-2. Gainesville, FL: The

Center for Research in Psy- Chophysiology. University of Florida.

Hauk, O. (2004). Keep it simple: a case for using classical minimum norm estimation in the analysis of EEG and MEG data. *NeuroImage*, *21*(4), 1612–1621.

McMahon, D. B. T., & Leopold, D. A. (2012). Stimulus Timing-Dependent Plasticity in High-Level Vision. *Current Biology*, *22*(4), 332–337. https://doi.org/10.1016/j.cub.2012.01.003

Roesmann, K., Kroker, T., Hein, S., Rehbein, M., Winker, C., Leehr, E. J., Klucken, T., & Junghöfer, M. (2022). Transcranial direct current stimulation of the ventromedial prefrontal cortex modulates perceptual and neural patterns of fear generalization. *Biological Psychiatry: Cognitive Neuroscience and Neuroimaging*, *7*(2), 210–220. https://doi.org/10.1016/j.bpsc.2021.08.001

Roesmann, K., Leehr, E. J., Böhnlein, J., Steinberg, C., Seeger, F., Schwarzmeier, H., Gathmann, B., Siminski, N., Herrmann, M. J., Dannlowski, U., Lueken, U., Klucken, T., Hilbert, K., Straube, T., & Junghöfer, M. (2022). Behavioral and Magnetoencephalographic Correlates of Fear Generalization are Associated with Responses to Later Virtual Reality Exposure Therapy in Spider Phobia. *Biological Psychiatry: Cognitive Neuroscience and Neuroimaging*, *7*(2), 221–230. https://doi.org/10.1101/2021.03.23.21253886

Roesmann, K., Wessing, I., Kraß, S., Leehr, E. J., Klucken, T., Straube, T., Junghöfer, M., Krass, S., Klucken, T., Straube, T., & Junghoefer, M. (2022). Developmental aspects of fear generalization – A MEG study on neurocognitive correlates in adolescents versus adults. *Developmental Cognitive Neuroscience*, *58*(October), 101169. https://doi.org/10.1016/j.dcn.2022.101169.

Schiele, M. A., Reinhard, J., Reif, A., Domschke, K., Romanos, M., Deckert, J., & Pauli, P. (2016). Developmental aspects of fear: Comparing the acquisition and generalization of conditioned fear in children and adults. *Developmental Psychobiology*, *58*(4), 471–481. https://doi.org/10.1002/dev.21393.

Tottenham, N., Tanaka, J.W., Leon, A.C., McCarry, T., Nurse, M., Hare, T.A., Marcus, D.J.,Westerlund, A., Casey, B.J., Nelson, C., 2009. The NimStim set of facial expres-sions: judgments from untrained research participants. Psychiatry Res. 168 (3),242–249.
